# Factors affecting outdoor physical activity in extreme temperatures in a sub-tropical Chinese urban population: an exploratory telephone survey

**DOI:** 10.1186/s12889-022-14788-0

**Published:** 2023-01-14

**Authors:** Janice Y. Ho, Holly Y. C. Lam, Zhe Huang, Sida Liu, William B. Goggins, Phoenix K. H. Mo, Emily Y. Y. Chan

**Affiliations:** 1 Jockey Club School of Public Health and Primary Care, The Chinese University of Hong Kong, Hong Kong, China; 2 Faculty of Medicine, National Heart & Lung Institute, Imperial College London, London, UK; 3 Collaborating Centre for Oxford University and CUHK for Disaster and Medical Humanitarian Response (CCOUC), Hong Kong, China; 4 GX Foundation, Hong Kong, China; 5 Nuffield Department of Medicine, University of Oxford, Oxford, UK

**Keywords:** Extreme temperature, Heat, Cold, Physical activity, Chronic disease, Climate change

## Abstract

**Background:**

Physical activity (PA) can be affected by extreme temperatures, however fewer studies have identified factors impacting this relationship. This study sought to identify factors associated with changes of outdoor PA during extreme cold/heat events in a sub-tropical Chinese urban population, including factors of sociodemographic, health conditions, temperature-related awareness and attitude, and protective behaviours.

**Methods:**

Two telephone surveys were conducted a week after extreme cold/heat events in 2016 and 2017 among a cohort of Hong Kong residents over age 15. Data was collected on self-reported changes in outdoor PA level during the periods of extreme temperatures, health status, comorbidities, sociodemographic, and temperature-related awareness, and behavioural variables. We conducted multivariable logistic regression analyses to assess predictors of change in outdoor PA over the two extreme temperature events.

Results and Conclusion: Among 435 participants (42.8% response rate), over a third of the participants reported decreased outdoor PA level in extreme temperature events, while 10% reported an increase in extreme heat. Self-reported cardiovascular diseases were associated with decreased PA level in extreme cold, while hypertension was associated with unchanged/increased PA level in extreme heat. These results suggest physical activity to be an important consideration in the understanding of climate change-and-health pathways and meriting further research.

**Supplementary Information:**

The online version contains supplementary material available at 10.1186/s12889-022-14788-0.

## Background

Physical activity can be affected by ambient temperatures [1, 2]. Cold temperatures are known to be a barrier to physical activity that reduce physical activity levels [3, 4]. In hot temperatures, the human body would not only face a physiological limit, but also voluntarily reduce the amount of work conducted to thermoregulate [5]. Studies globally demonstrate an overall ‘inverse U-shaped’ association between temperature and physical activity with reduced physical activity levels at both low and high temperatures, although the threshold depends on the season and location [6–8]. This can be seen across a wide variety of physical activities measurements, ranging from pedometers and accelerometers [9–12], to bike share usage [13, 14], hiking and trail observations [15, 16], and survey questionnaires [17]. However, fewer studies have sought to identify what factors impact how extreme temperatures affect physical activity.

People living with chronic non-communicable diseases (NCDs) are more vulnerable to extreme temperatures and at greater risk of temperature-related mortality and morbidity [18, 19], as extreme temperatures of cold and heat add additional stress to cardiovascular and respiratory systems [20, 21]. Several patient-specific studies have assessed temperature effects on physical activity among those with NCDs, such as arthritis or chronic obstructive pulmonary disease (COPD) [3, 22, 23]. These studies demonstrate the possible variations between different NCDs in their physical activity response to extreme temperatures. However, temperature-physical activity studies in the general population have rarely adjusted for health-related predictors such as self-reported health status and NCDs [2, 24]. Even fewer studies have sought to differentiate between multiple NCDs in prior temperature-physical activity studies. More must be understood about the health conditions and different chronic NCDs that influence physical activity during extreme temperatures in the general population.

Previous studies on temperature and physical activity have additionally not assessed the effects of temperature-related awareness and attitudes, and the influence of protective behaviours. These are often studied in behavioural responses to extreme temperature warning systems [25–29], and pose as important mediating pathways to understand people’s perceived risk and response to extreme temperatures. A person’s perception may inform and influence their behaviour, including the behaviour of physical activity. As such, the inclusion of these indicators may increase our understanding on whether participants choose to conduct physical activity in extreme temperatures.

This study aims to identify factors influencing outdoor physical activity response during extreme cold and heat events in a subtropical city. Factors explored include sociodemographic characteristics, health status and chronic NCDs, temperature-related awareness and attitude, and protective behaviours.

## Methods

### Study setting

Hong Kong is a sub-tropical Chinese city which experiences average monthly mean temperatures ranging between 16–29 °C [30]. During periods of extreme temperatures, the local meteorological authority, the Hong Kong Observatory, issues warnings to alert the public and relevant government departments to take preventive measures [31]. The Cold Weather Warning (CWW) is hoisted when the temperatures drop below 12 °C or when the Weather Stress Index is below the 2.5^th^ percentile [32]. The Very Hot Weather Warning (VHWW), on the other hand, is hoisted when the measurements cross 30.5 °C on the Hong Kong Heat Index (HKHI). The HKHI is an index developed specifically for the hot and humid subtropical climate of the city and based on a combination of natural wet bulb temperature (a thermometer covered by wetted wick to measure combined effects of humidity, wind, solar radiation and temperature), globe temperature (a thermometer within a black globe to measure combined effects of temperature, solar radiation and wind), and dry bulb temperature (ambient air temperature) [33].

In this study, the study periods were defined as the time from the issuing of a particular Cold Weather Warning/Very Hot Weather Warning until the end of the survey administration period.

### Environmental variables

Daily meteorological and air pollution variables were obtained for the study period and prior months from the Hong Kong Observatory (HKO) and Environmental Protection Department, respectively. Daily meteorological variables were taken from the HKO meteorological station located at the city center, and included mean pressure, temperature (maximum, mean, minimum, dewpoint), relative humidity, cloud cover, rainfall, sunshine hours, windspeed and wind direction. Air pollution variables were retrieved at the hourly level across all 14 general monitoring stations, except Tap Mun, and aggregated to a daily average. These included CO, NO2, NOx, O3, SO2, Respirable Suspended Particulates (PM10) and Fine Suspended Particulates (PM2.5). Records from Tap Mun were excluded due to its remote island location and small population.

### Survey data collection

A repeated measures cohort study with two population-based telephone surveys were conducted in 2016 and 2017. A similar survey questionnaire was used over the two-year study, with the main differences related to the seasonal time point of the survey. This analysis was limited to participants that answered in both surveys. The first telephone survey was conducted a week after the hoisting of a Cold Weather Warning on 21–27 January 2016. The second survey was conducted a week after the hoisting of a Very Hot Weather Warning on 25–30 July 2017. These two study periods will be hereafter known as the ‘2016 extreme cold’ and ‘2017 extreme heat’, respectively. The study design was further described elsewhere [34–36]. In brief, the telephone survey used a Random Digit Dialing method to randomize the household landline telephone numbers sampled from each of Hong Kong’s 18 districts (covering 94.24% of Hong Kong households in 2016, [37]). Selection of the eligible participant within each household was further randomized using the ‘last birthday method’, whereby the eligible household member with the most recent birthday was asked to participate in the survey. The target population of this study was all Cantonese-speaking non-institutionalized Hong Kong residents over age 15, as 94.6% of the Hong Kong population regularly speak or are able to speak Cantonese [38]. To collect adequate representation of the working population, calls were made from 6:30 pm to 10:00 pm on weekdays, and during the daytime on weekends.

At the end of the 2016 survey, participants were asked to provide their phone number if they were willing to participate in the follow-up survey of the study. The recorded number was used to contact the same participant in the 2017 follow-up survey. At least five attempts were made to reach the participant before they were considered “lost-to-follow-up”. All interviews were administered by trained interviewers.

Ethics approval for this study was obtained from the Survey and Behavioural Research Ethics Committee of The Chinese University of Hong Kong. All methods were carried out in accordance with relevant guidelines and regulations. Verbal informed consent was obtained from all participants prior to the start of each survey. Verbal informed consent is approved by the ethics committee (Survey and Behavioural Research Ethics Committee of The Chinese University of Hong Kong).

### Variables

The survey measures were based on previously published studies that examined self-reported health outcomes in the subtropical urban population [39, 40]. The main outcome of physical activity was assessed through the following question: “**Since {date Cold Weather Warning/Very Hot Weather Warning was hoisted} till today, have you increased, decreased, or remained the same in the amount of outdoor physical activity**?”. The survey questionnaire also collected potential effect modifying factors of (1) sociodemographic characteristics (including gender, age, education, district, income, occupation, marital status, living alone, housing, and home ownership), (2) health conditions (including chronic NCDs, general self-rated health, seasonal self-rated health, recent seeking of medical treatment, and usage of long-term medications), (3) temperature-related awareness and attitudes (including Awareness of CWW/VHWW, Knowledge of today’s min/max temperature, Agree cold/hot weather impacts health, Agree the health impacts of cold/hot weather can be avoided, and Agree I have adequate knowledge to handle the health impact of cold/hot weather), and (4) protective behaviours (including Avoid prolonged exposure to cold winds/avoid staying out in the sun, Use heating devices/AC, and a sum measure of other protective behaviours). Further details of the survey questions and categorizations can be found in Supplemental Materials Table S1. The 2017 survey responses were chosen for variables assessed at both survey timepoints (such as age). A pilot study (*n* = 53) was administered to test the reliability of the survey in December 2015.

Compared to the initial sample in 2016, the follow-up sample in 2017 had a slightly different age distribution, as there was more lost-to-follow-up among working adults aged 25–44. Other demographic factors remained comparable between the initial and follow-up samples. The final study sample (*n* = 435) was representative of the general population in gender, district regions, marital status, and household income, but tended to be older and more well-educated (see Table 1). To account for these differences, the multivariable analyses were adjusted for gender, age, and education.

**Table 1:** Demographic comparison between the telephone survey cohort and Hong Kong general population

Demographics	Initial 2016 Survey		Follow-up 2017 survey		Population 2016 Census		Follow-up vs. Census *p*-value^	
	n	%	n	%	%	Derived n		
Gender	*N* = 1017		*N* = 435					
Male	437	43	200	46	47.6	207.06	0.498	
Female	580	57	235	54	52.4	227.94		
Age	*N* = 1017		*N* = 435					
15–24	126	12.4	65	14.9	12.6	54.81	< 0.001	**
25–44	315	31	101	23.2	31.8	138.33		
45–64	384	37.8	168	38.6	36.8	160.08		
≥ 65	192	18.9	101	23.2	18.8	81.78		
Region	*N* = 1015		*N* = 435					
Kowloon	315	31	140	32.2	30.8	133.98	0.818	
Hong Kong Island	182	17.9	70	16.1	16.6	72.21		
New Territories	518	51	225	51.7	52.6	228.81		
Marital Status	*N* = 1012		*N* = 435					
Single	330	32.6	147	33.8	30.1	130.94	0.223	
Married	602	59.5	243	55.9	58.3	253.61		
Separated/ divorced	80	7.9	45	10.3	11.6	50.46		
Household Income (HKD)	*N* = 945		*N* = 407					
$40,000 +	317	33.5	132	32.4	30.8	125.36	0.069	
$20,000-$39,999	333	35.2	129	31.7	27.9	113.55		
< $20,000	295	31.2	146	35.9	41.3	168.09		
Education	*N* = 1015		*N* = 434					
Post-secondary	377	37.1	154	35.5	33.2	144.09	< 0.001	**
Secondary	501	49.4	222	51.2	46.2	200.51		
Primary or below	137	13.5	58	13.4	20.6	89.40		

Data obtained from telephone survey cohort conducted in Hong Kong, 2016–2017

^Chi-square test was used to measure the overall difference in demographic proportions between this study and the 2016 Hong Kong Population Census [38]. Census numbers excluded foreign domestic helpers and those under age 15. District region census numbers were calculated from [41]

^*^p-value ≤ 0.05 **p-value ≤ 0.01

### Statistical analysis

Descriptive statistics on the sociodemographic characteristics, physical activity responses, and health conditions were reported. T-tests were used to confirm the difference in environmental variables between the study periods and the preceding days in the study period month.

The main outcome variable of physical activity (PA) was transformed into three separate binary outcomes: 1) Decreased outdoor PA in extreme cold vs. no change/increased outdoor PA; 2) Decreased outdoor PA in extreme heat (2017) vs. no change/increased outdoor PA; and 3) Increased outdoor PA in either extreme temperature event vs. decreased/no change outdoor PA. Potential factors of sociodemographic characteristics, health conditions, temperature-related awareness and attitudes, and protective behaviours were first screened individually on their relationship with each physical activity outcome using Chi-squared test. Variables with p < 0.25 in the bivariate analyses were then included in the subsequent analysis.

Separate multivariable forward stepwise logistic regression models were conducted for the three binary PA outcomes to identify factors of changing outdoor PA level in extreme temperatures, adjusted for age, gender, and education. The forward stepwise regression model was chosen for this exploratory study because of the number of potential variables – many that have not been considered in extreme temperature physical activity studies before. This method allows the consideration of models with different combinations of variables and is reproducible. When we tested for biviarate correlations, our benchmark was set for p < 0.25 for inclusion in the forward stepwise logistic regression model. This enabled the initial elimination from a wide range of potential variables for the multivariable model. Furthermore, sensitivity analyses were conducted to test the robustness of variable selection using two methods: generalized linear models and multi-model inference using the MuMIn() package [42].

Statistical significance was set at *p* ≤ 0.05. All statistical tests were conducted with IBM SPSS Statistics for Windows, Version 20.0. [43], apart from the sensitivity analyses which used the statistical software R (version 4.1.3) [44].

## Results

The study periods were January 21 – February 4, 2016 for the 2016 extreme cold, and July 28 – August 13, 2017 for the 2017 extreme heat. In the 2016 extreme cold survey, a total of 1,017 successfully completed the interview (response rate 1,017/1,598 = 63.6%), of which 436 participants were successfully followed-up during the 2017 extreme heat (response rate = 42.87%). One participant was further excluded from analysis due to missing data on the main outcome of interest, resulting in a final sample size of 435 participants.

Overall, a large proportion of respondents reported a decrease in outdoor physical activity during the 2016 extreme cold (41.6%) and the 2017 extreme heat episodes (35.2%) (see Table 2). There was a significantly greater proportion of respondents reporting a decrease in outdoor PA level during the 2016 extreme cold compared to the 2017 extreme heat (*p* = 0.029, McNemar’s test). Increased outdoor physical activity, which was reported among 10.3% of participants across either extreme temperature, was significantly greater in extreme heat (9.2%) than extreme cold (*p* ≤ 0.001, McNemar’s test). When PA responses were compared across the extreme temperature events, 36.3% of the participants reported to maintain their original level of outdoor physical activity during both the extreme temperature periods, while 20.7% reported decreased outdoor physical activity in both periods (see Table 2).

**Table 2:** Comparison of changes in outdoor physical activity across 2016 extreme cold and 2017 extreme heat (*N* = 435)

	**2016 extreme cold**			
2017 extreme heat	Increase	No Change	Decrease	Total
Increase	5 (1.1%)	25 (5.7%)	10 (2.3%)	40 (9.2%)
No change	3 (0.7%)	158 (36.3%)	81 (18.6%)	242 (55.6%)
Decrease	2 (0.5%)	61 (14.0%)	90 (20.7%)	153 (35.2%)
Total	10 (2.3%)	244 (56.1%)	181 (41.6%)	435 (100%)

Data obtained from telephone survey cohort conducted in Hong Kong, 2016–2017

In terms of health-related conditions, 141 participants (32.6%) reported having chronic NCDs. Of those, 55 participants reported having two or more chronic NCDs. The top five chronic NCDs reported were hypertension (15.4%), diabetes (8.0%), cardiovascular disease (5.1%), hypercholesterolemia (4.1%), and chronic pain (2.5%) such as arthritis. A total of 148 participants (34.0%) reported taking long-term medications. Most participants self-reported having normal to very good health, while 4.8% reported having bad health (see Table 3). A majority of participants reported an unchanged health status during the winter season (69.9%) and summer season (77.0%). However, 22.5% and 12.2% of participants reported worsened health status during the winter and summer seasons, respectively. During the extreme temperature events, 15.4% of participants sought medical treatment due to acute symptoms potentially related to extreme cold and heat.

**Table 3:** Responses on self-rated health status from the telephone survey cohort

	**General health**		**Seasonal health**	**Winter (2016)**	**Summer (2017)**
Very good	74 (17.0%)		Better	33 (7.6%)	47 (10.8%)
Good	141 (32.4%)		Same	304 (69.9%)	335 (77.0%)
Normal	199 (45.7%)		Worse	93 (22.5%)	53 (12.2%)
Bad	21 (4.8%)				

Data obtained from telephone survey cohort conducted in Hong Kong, 2016–2017

### Comparison of environmental conditions during extreme temperature events against preceding days

During the 2016 study period, the Cold Weather Warning (CWW) was hoisted for a cumulative amount of 243 h and 35 min, or 10.15 days over the 15-day study period. It included the coldest day since 1957, which had the 6^th^ lowest ever recorded minimum temperature of 3.1 °C during the afternoon of January 24, 2016 [45]. In the 2017 study period, the Very Hot Weather Warning (VHWW) was hoisted for a cumulative amount of 226 h and 45 min, or 9.45 days over the 17-day study period. The 2017 study period saw one of the highest daily mean temperatures for July on record, 31.8 °C on July 30, 2017 [46]. The maximum, mean and minimum temperatures during the study periods were statistically different from those in the preceding periods (lower than the preceding period in 2016 and higher in 2017, see Table 4). In addition, most air pollutants were significantly higher levels during the extreme heat period.

**Table 4:** Comparison of meteorological variables and air pollutants between 2016 and 2017 study periods and prior months, T-test

	**2016 Prior Days in Jan (*N* = 20)**	**2016 Extreme Cold (*N* = 15)**	**T-test**		**2017 Prior Days in Jul (*N* = 27)**	**2017 Extreme Heat (*N* = 17)**	**T-test**	
*Meteorological*	*Mean (S.D.)*	*Mean (S.D.)*	*p-value*		*Mean (S.D.)*	*Mean (S.D.)*	*p-value*	
Mean Pressure	1018.78 (3.22)	1023.25 (5.19)	.004	**	1007.9 (2.02)	1004.01 (3.74)	.001	**
Max Temp	19.54 (2.12)	14.75 (3.54)	< .0005	**	31.04 (1.82)	32.37 (1.53)	.017	*
Mean Temp	17.82 (1.77)	12.73 (3.75)	< .0005	**	28.37 (1.22)	29.94 (1.02)	< .0005	**
Min Temp	16.38 (1.93)	10.91 (4.16)	< .0005	**	26.57 (1.07)	27.93 (1.26)	< .0005	**
Dewpoint Temp	15.1 (2.35)	9.19 (6.49)	.004	**	25.46 (0.35)	25.85 (0.54)	.005	**
Rel. Humidity	84.4 (7.69)	80.53 (15.68)	.390		84.74 (5.71)	79.06 (4.62)	.001	**
Cloud cover	74.1 (21.2)	85.53 (21.59)	.127		80.11 (9.23)	73.88 (13.04)	.071	
Rainfall	8.52 (14.55)	7.2 (13.17)	.784		21.11 (42.37)	8.55 (16.25)	.250	
Sunshine Hrs	2.64 (3.23)	1.59 (3.38)	.356		4.84 (3.53)	6.29 (3.76)	.203	
Wind Speed	26.82 (10.84)	30.92 (13.17)	.320		21.5 (6.15)	22.82 (9.36)	.576	
Wind Direction (South = 0)	131.50 (18.14)	144.00 (18.44)	.053		72.59 (40.44)	65.88 (28.08)	.553	
*Air pollutants*								
Mean CO	99.52 (10.39)	85.07 (21.51)	.027	*	47.24 (3.25)	56.12 (12.1)	.009	**
Mean NO_2_	50.14 (11.21)	48.01 (14.23)	.623		25.18 (4.62)	36.05 (12.8)	.003	**
Mean NO_X_	81.41 (26.65)	95.22 (39.19)	.223		49.96 (12.87)	60.51 (14.73)	.016	*
Mean O_3_	37.07 (15.46)	24.99 (15.26)	.028	*	21.71 (3.9)	38.57 (24.61)	.012	*
Mean SO_2_	7.49 (2.13)	7.38 (2.4)	.887		5.34 (1.21)	8.37 (2.9)	.001	**
Mean RSP^a^	39.89 (18.35)	30.07 (14.67)	.098		12.89 (2.22)	26.78 (16.39)	.003	**
Mean FSP^a^	29.07 (12.91)	20.52 (9.27)	.037	*	7.1 (1.51)	16.44 (12.68)	.008	**

Data obtained from Hong Kong Observatory and Environmental Protection Department, 2016–2017

^*^p-value ≤ 0.05

^**^p-value ≤ 0.01

^a^*RSP* Respirable Suspended Particulates; *FSP* Fine Suspended Particulates

### Multivariable logistic regression models

All variables that showed an association (*p* < 0.25) with the physical activity outcomes in the bivariate analyses were entered into multivariable regression models. The results of the bivariate analyses are listed in Supplemental Materials, Table S2.

**Fig. 1:**
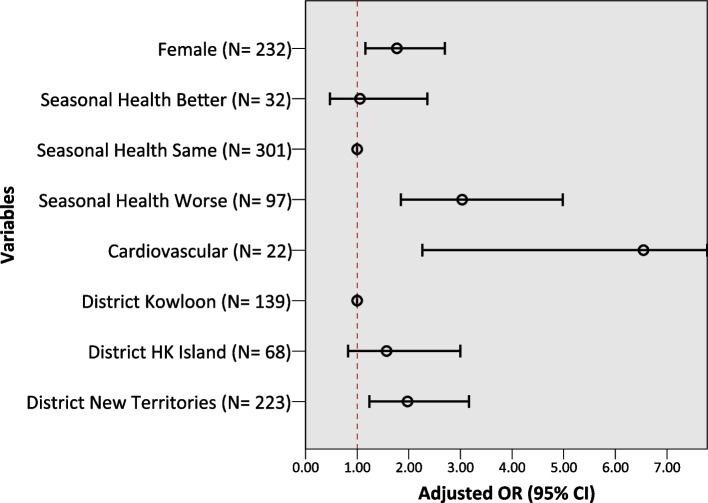
Multivariable logistic regression results for decreased outdoor PA, 2016 extreme cold. Final model: 178 reported decreased outdoor PA, *N* = 430, Predicted 66.3%, Nagelkerke R^2^ 0.155

During the 2016 extreme cold, self-reported decreased outdoor physical activity was associated with a greater likelihood of being female (Adjusted Odds Ratio (AOR) = 1.77, 95% confidence interval (CI): 1.16–2.70), living in the more suburban region of the New Territories (AOR = 1.98, 95% CI: 1.23–3.17; vs. Kowloon), worsened health in the winter season (AOR = 3.03, 95% CI: 1.85–4.98; vs. unchanged health status in the winter), and those with cardiovascular disease (AOR = 6.55, 95% CI: 2.26–18.94) (see Fig. 1 and Table S3 in the Supplemental Materials for full model details).

**Fig. 2:**
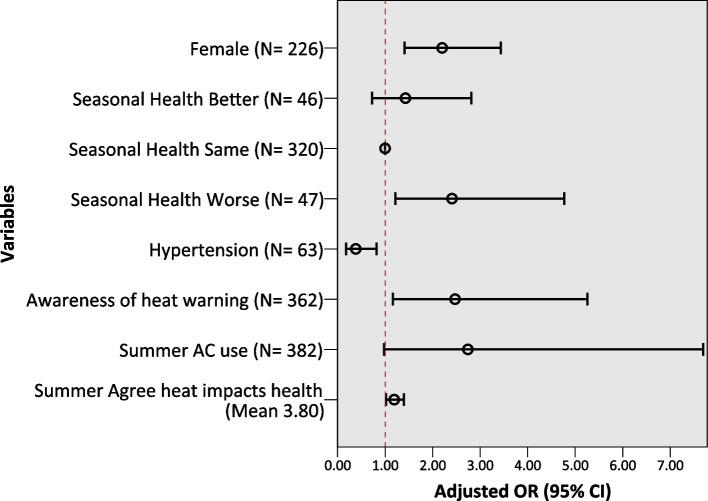
Multivariable logistic regression results for decreased outdoor PA, 2017 extreme heat. Final model: 147 reported decreased outdoor PA, *N* = 413, Predicted 67.3%, Nagelkerke R^2^ 0.165

During the 2017 extreme heat, self-reported decreased outdoor physical activity was associated with a greater likelihood of being female (AOR = 2.20, 95% CI: 1.41–3.44), self-reported worsened health in the summer (AOR = 2.41, 95% CI: 1.21–4.77; vs. unchanged health status in the summer), awareness of VHWW (AOR = 2.47, 95% CI: 1.16–5.26), and agreeing that heat impacts health (AOR = 1.19, 95% CI: 1.02–1.40), while those with hypertension were associated with a lesser likelihood of decreased outdoor PA levels (AOR = 0.38, 95% CI: 0.18–0.82). Using AC remained in the final model but was slightly non-statistically significant (AOR = 2.74, 95% CI: 0.98–7.69) (see Fig. 2 and Table S4 in the Supplemental Materials for full model details).

**Fig. 3:**
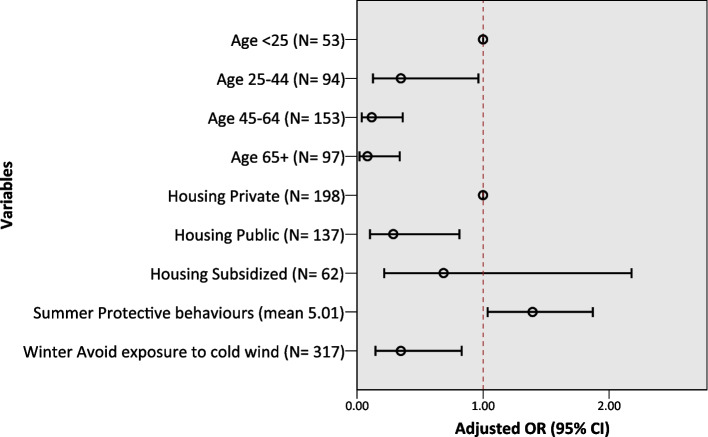
Multivariable logistic regression results for increased outdoor PA in either extreme temperature event. Final model: 31 reported increased outdoor PA, *N* = 397, Predicted 92.7%, Nagelkerke R^2^ 0.194 (Education was excluded from the model since there were zero cases of increased physical activity among those with primary education or below.)

Self-reported increased outdoor physical activity during either extreme temperature event was more likely to be observed among those under 25 (vs. aged 25–44 AOR = 0.35, 95% CI: 0.13–0.96; aged 45–64 AOR = 0.12, 95% CI: 0.04–0.36; aged 65 and above AOR = 0.08, 95% CI: 0.02–0.34), those in private housing (vs. public housing AOR = 0.29, 95% CI: 0.10–0.81), and those conducting protective behaviours during the extreme heat (AOR = 1.39, 95% CI: 1.04–1.87), but was less likely to be observed among those avoiding exposure to cold winds (AOR = 0.35, 95% CI: 0.15–0.83) (see Fig. 3 and Table S5 in the Supplemental Materials for full model details).

### Sensitivity analysis

Sensitivity analyses using generalized linear models and multi-model inference both demonstrated the robustness of the multivariable regression models and variable selection. The results can be seen in Supplemental Materials Tables S6-S8.

## Discussion

During extreme temperature events in subtropical Hong Kong, our study found over a third of the participants reported a decrease in outdoor physical activity in either extreme cold (41.6%) or extreme heat (35.2%), while 9.2% reported an increase in physical activity in extreme heat. Overall, our research found a net decrease in outdoor physical activity levels during extreme temperatures in a subtropical setting, similar to previous studies located in Canada [9]. However, extreme cold led to a slightly greater proportion of reported decreased outdoor physical activity compared with extreme heat. The study findings demonstrate that even in a sub-tropical climate like Hong Kong (Koppen-Geiger climate classification: Cwa), extreme cold has a substantial effect on physical activity behaviour. Previous research in sub-tropical settings also identified cold temperature effects on mortality, particularly finding the cold temperature effect to be larger in warmer cities [47, 48]. Meanwhile, the prevalence of increased outdoor physical activity was found greater in extreme heat rather than extreme cold. However, heat still poses a health risk even to the young or physically active populations, as people of all ages including relatively young adults, have occasionally died in Hong Kong while conducting vigorous outdoor physical activity in the heat, such as hiking [49, 50].

The findings of this study demonstrate different response behaviours to extreme temperatures among chronic disease patients. Among chronic NCDs, our study identified cardiovascular disease to be associated with decreased physical activity in extreme cold, and its related risk factor, hypertension, to be associated with unchanged or increasing PA level in extreme heat. Previous temperature-physical activity studies have mostly assessed those with COPD or arthritis, while seldomly addressing those with cardiovascular diseases. However, our study found that in extreme cold, those with cardiovascular disease were associated with a 6.5 times likelihood of decreasing physical activity among the chronic NCDs. This was aligned with mortality research that found up to 70% of excess winter deaths were cardiovascular-related [51] and demonstrated an increased risk of cardiovascular markers in cold temperatures [52–54]. Our findings indicate that the study population was aware of the cold-related risks of cardiovascular disease or instinctively decreased their outdoor physical activity levels in efforts to avoid the cold. However, other NCDs were not associated with change in PA levels in extreme cold, suggesting a low awareness on cold-related risks to their chronic conditions. This is quite dangerous for those with hypertension or chronic respiratory disease, such as COPD and asthma, who have higher health risks in low temperatures [55–58].

In extreme heat, this study found that those with hypertension were less likely to decrease outdoor physical activity in extreme heat when compared to those without hypertension. This is in contrast to a previous small-scale study in Germany which found reduced physical activity in hot temperatures above 25 °C among 15 hypertensive patients [59]. The effects of hypertension on exercise in hot conditions are still unclear from physiological studies [24, 60]. Several studies have found lower blood pressure and better hypertension control during summer season and high temperatures [61–64]. However, at the same time, hypertensive patients are at an increased risk of heat-related complications during exercise [65]. Lower skin blood flow and less core-to skin heat transfer was found among those with hypertension during exercise-induced heat stress [24], suggesting that the body’s thermoregulatory function was impaired because of “structural and functional alterations”. The use of anti-hypertensive medications may further alter the thermoregulatory response to heat. Thus, our study findings suggest that hypertensive persons may feel quite manageable to conduct physical activity in hot weather while overlooking their actual risk posed by the heat. It may be critical that hypertension patients are informed of the increased heat-related risks of conducting PA in hot days. Meanwhile, other NCDs found no significant association with change in PA levels in extreme heat. Yet, ischemic heart disease and diabetes patients are highly vulnerable in high temperatures, and they should be aware of their heat-related risks [66–68].

Decreased physical activity in extreme temperatures could inhibit physical activity and accelerate the deterioration and adverse outcomes of chronic disease patients in the long run. It is known that regular physical activity is essential to the disease management of chronic disease patients. Physical activity interventions have been shown to improve the risk markers and survival outcomes for cardiovascular disease, type 2 diabetes, and selected cancers [69]. Previous studies have also found that winter outdoor physical activity was associated with lower winter mortality [70], and regular exercise was necessary to reduce the effect of cold on physiological changes for ischaemic heart disease patients [71]. Hence, specific efforts should be made to enquire about patients’ physical activity habits to ensure their exercise programmes [72] can be feasibly sustained in extreme temperatures. In order to reduce risk of extreme heat or cold exposures, recommendations could be made to diversify physical activity options or schedules, whether it is conducting more indoor activity or finding appropriate times of the day to conduct outdoor activities. Government entities and public sport facilities could also encourage the general population to conduct physical activity in the extreme cold, while opening up more accessible indoor opportunities in extreme heat.

In terms of temperature-related factors and protective behaviours, no associations were found with decreased outdoor PA during extreme cold. In contrast, during extreme heat, decreased outdoor PA was associated two temperature-related factors (awareness of heat warning and agree that heat impacts health) and marginally associated with protective behaviour of AC use. Increased outdoor PA was associated with conducting more summer protective behaviours, and less likelihood to avoid exposure to cold winds. The association with summer protective behaviours suggest that the increase in outdoor PA during extreme heat may be part of a health-conscious decision rather than a rash one.

As climate change will lead to an increase in frequency and severity of extreme temperatures, particularly heatwaves, the association of NCDs and physical activity in extreme temperatures merits further investigation. Whether or not there is additional health risk as a result of those who maintain or increase their outdoor physical activity in extreme heat, and whether decreased outdoor physical activity during extreme temperatures will have implications on long-term health needs to be understood. Further analyses including information on baseline physical activity levels and substitution effect of indoor physical activity may enhance the understanding between physical activity and extreme temperatures, and help to improve relevant governmental, clinical, and infrastructural considerations in response to extreme temperatures.

### Strengths and limitations

The strengths of this study included a repeated measures study design which followed the same participants during both the extreme hot and cold temperature events. The short study periods and the one-week lag between the onset of the temperature events and data collection periods helped to reduce any potential recall bias of participants self-reported outdoor physical activity. However, the study findings were unable to determine a causal direction in the associations between physical activity and health under extreme temperatures. The survey also did not measure baseline physical activity levels or quantify the change of frequency and intensity of physical activity during extreme temperatures. The validity of the physical activity measure has not been assessed. As our study focuses on outdoor physical activity, it is not understood whether the respondents had subsequently substituted outdoor physical activity for indoor physical activity. Finally, the study experienced a loss to follow-up, leading to a smaller final sample size. Low statistical power due to a small sample size may have limited our ability to discover associations with different chronic disease groups. Further research could assess more chronic disease participants in their physical activity behaviour during extreme temperatures. Additionally, the small sample size may have implications on the generalizability of the study findings, and they should be interpreted with caution.

Our study results were unable to tease out the effect of air pollution compared with the extreme temperature event, as air pollutants were also significantly higher during the extreme heat period. Air pollution is suggested to decrease physical activity levels in periods of high pollution; however, the evidence is still relatively sparse, particularly for outdoor physical activity [73].

## Conclusions

Our study demonstrates that outdoor physical activity decreases in extreme temperatures in over a third of the participants in a subtropical urban population. A greater proportion of participants’ physical activity were affected in extreme cold rather than extreme heat. Those with cardiovascular disease were more likely to decrease physical activity in extreme cold, while those with hypertension were less likely to decrease physical activity in extreme heat. Some protective behaviours and temperature-related awareness and attitudes were associated with change in physical activity levels, particularly during extreme heat. Healthcare providers should provide guidance to patients on the potential risks of conducting physical activity levels in extreme temperatures, particularly those with hypertension.

## Supplementary Information

**Additional file 1: Table S1.** Details of included survey variables. **Table S2.** Bivariate analysis for change in PA during extreme temperatures (Chi-squared tests and T-tests). **Table S3.** Multivariable logistic regression for Decreased outdoor PA in 2016 extreme cold, full model results. **Table S4.** Multivariable logistic regression for Decreased outdoor PA in 2017 extreme heat, full model results. **Table S5.** Multivariable logistic regression for Increased outdoor PA in either extreme temperature, full model results. **Table S6.** Sensitivity analysis for Decreased outdoor PA in 2016 extreme cold. **Table S7.** Sensitivity analysis for Decreased outdoor PA in 2017 extreme heat. **Table S8.** Sensitivity analysis for Increased outdoor PA in either extreme temperature event.

## Data Availability

The datasets used and/or analysed during the current study are available from the corresponding author on reasonable request.

## Abbreviations

PA: Physical activity
WHO: World Health Organization
NCDs: Non-communicable diseases
CWW: Cold Weather Warning
VHWW: Very Hot Weather Warning
AOR: Adjusted Odds Ratio
CI: Confidence interval
