# Supplementary material for: Factors affecting outdoor physical activity in extreme temperatures in a sub-tropical Chinese urban population: an exploratory telephone survey
Source: BMC Public Health. 2023 Jan 14;23:101. doi: 10.1186/s12889-022-14788-0 (PMC9840260; doi:10.1186/s12889-022-14788-0)
Supplement: Supplementary file 1 — Additional file 1: Table S1. Details of included survey variables. Table S2. Bivariate analysis for change in PA during extreme temperatures (Chi-squared tests and T-tests). Table S3. Multivariable logistic regression for Decreased outdoor PA in 2016 extreme cold, full model results. Table S4. Multivariable logistic regression for Decreased outdoor PA in 2017 extreme heat, full model results. Table S5. Multivariable logistic regression for Increased outdoor PA in either extreme temperature, full model results. Table S6. Sensitivity analysis for Decreased outdoor PA in 2016 extreme cold. Table S7. Sensitivity analysis for Decreased outdoor PA in 2017 extreme heat. Table S8. Sensitivity analysis for Increased outdoor PA in either extreme temperature event. [file 12889_2022_14788_MOESM1_ESM.docx]

**Title: Impact of cold and hot extreme temperatures on outdoor physical activity in a sub-tropical Chinese urban population: A telephone survey**

Authors: Janice Y. Ho, Holly Y.C. Lam, Zhe Huang, Sida Liu, William B. Goggins, Phoenix K.H. Mo, Emily Y. Y. Chan

Supplemental materials: Table S1-8

|  | Page number |
| --- | --- |
| Table S1. Details of included survey variables | 2 |
| Table S2. Bivariate analysis for change in PA during extreme temperatures (Chi-squared tests and T-tests) | 5 |
| Table S3. Multivariable logistic regression for Decreased outdoor PA in 2016 extreme cold, full model results | 8 |
| Table S4. Multivariable logistic regression for Decreased outdoor PA in 2017 extreme heat, full model results | 9 |
| Table S5. Multivariable logistic regression for Increased outdoor PA in either extreme temperature, full model results | 10 |
| Table S6. Sensitivity analysis for Decreased outdoor PA in 2016 extreme cold | 11 |
| Table S7. Sensitivity analysis for Decreased outdoor PA in 2017 extreme heat | 12 |
| Table S8. Sensitivity analysis for Increased outdoor PA in either extreme temperature event | 13 |

**Table S1. Details of included survey variables**

| Variables | Survey response options | Categorization for analysis |
| --- | --- | --- |
| Outdoor physical activity  “Since {date Cold Weather Warning/Very Hot Weather Warning was hoisted} till today, have you increased, decreased, or remained the same in the amount of outdoor physical activity?” | Increased/Decreased/No change/ Don’t know | 1) Decreased in extreme cold/ No change or increased  2) Decreased in extreme heat/ No change or increased  3) Increased in either temperature event/ Decreased or no change |
| ***Sociodemographic characteristics*** | | |
| Gender | Male/Female | |
| Age | 15-19/20-24/25-29/30-34/35-39/40-44/45-49/50-54/55-59/60-64/65-69/≥70 | 15-24/ 25-44 /45-64 /≥65 |
| Education | No formal education/ Primary/ Junior high school/ High school / Foundation / Diploma / Higher Diploma / Associate Degree / Bachelor’s Degree/ Master’s degree or above | Primary or below/ Secondary/ Post-secondary |
| District | (18 districts) Central & Western/ Wan Chai/ Eastern/ Southern/ Yau Tsim Mong/ Sham Shui Po/ Kowloon City/ Wong Tai Sin/ Kwun Tong/ Kwai Tsing/ Tsuen Wan/ Tuen Mun/ Yuen Long/ North/ Tai Po/ Shatin/ Sai Kung/ Islands | (3 regions) Hong Kong Island/Kowloon/ New Territories |
| Income (monthly household income) | <$2000/ $2000-3999 / $4000-5999 / $6000-7999/ $ 8000-9999 / $10000-14999 / $15000-19999 / $20000-24999  / $25000-29999 / $30000-39999 / >$40000/ Don’t know | <$20,000/ $20,000-$39,999/ $40,000+ |
| Occupation | Managers and administrators / Professionals / Associate professionals / Clerical support workers / Service and sales workers/ Craft and related workers / Plant and machine operators and assemblers/ Elementary occupations/ Skilled agricultural and fishery workers/ Housewife / Student / Unemployed / Retired / Other | Professional or clerical / Service / Blue collar / Housewife / Student / Unemployed or retired |
| Marital status | Unmarried/ Cohabitation/ Married / Separated or divorced/Widowed | Single/ Married or cohabitation/ Separated, divorced, or widowed |
| Living alone | “Including yourself, how many people (including servants) live with you in your house” - open-ended response | 1 |
| Housing | Public rental / Subsidized home ownership / Private buildings/ Private village house/Non-residential housing/ Temporary housing | Public/ Subsidized / Private |
| Home ownership | Own / Rent/ Shared rental / Other | Owned / Rent |
| ***Health-related*** | | |
| General self-rated health | Excellent/Very good/Good/Normal/Bad | Excellent to very good / Good / Normal/ Bad |
| Seasonal self-rated health | Much worse/ Relatively worse/ Similar / Relatively better | Worse / Same/ Better |
| Sought medical treatment | Yes/No | |
| Long-term medications | Yes/No | |
| Chronic NCDs | None/ Cardiovascular disease/ Diabetes/ Hypertension/ Stroke/ Kidney disease/ Liver disease/ Psychological disease/ Respiratory disease /Chronic pain (e.g. arthritis) / Cancer / Eye disease / Brain / Gynecology/ Gastrointestinal disease/ Thyroid disease / High cholesterol / High blood sugar | |
| ***Temperature-related awareness and attitude*** | | |
| Awareness of CWW/ VHWW | Yes/No/Don’t know | Yes/No |
| Knowledge of today’s min/max temperature | Yes/No | |
| Agree cold/hot weather impacts health | Scale of 1-6 from Completely disagree to completely agree | |
| Agree the health impacts of cold/hot weather can be avoided | Scale of 1-6 from Completely disagree to completely agree | |
| Agree I have adequate knowledge to handle the health impact of cold/hot weather | Scale of 1-6 from Completely disagree to completely agree | |
| ***Protective behaviours*** | | |
| Have you performed the following actions between last week until today? | Yes/No | Avoid prolonged exposure to cold winds/ avoid staying out in the sun |
|  |  | Use heating devices/AC |
|  |  | Sum of the following behaviors* (wear suitable clothes, use sunscreen, drink more (warm) water, pay attention to weather information, maintain indoor ventilation, pay attention to elderly, and to young children) |

Variables included from telephone survey cohort conducted in Hong Kong, 2016-2017

“Don’t know” responses were treated as missing and removed from further analyses. “Other” responses were assessed for their textual response and combined with an appropriate group, or else treated as a missing response and removed from further analysis.

*The sum measure used as a proxy to assess if compliance with overall heat or cold-related protective behaviours is associated with participants’ outdoor physical activity. All protective behaviours were taken from the recommended guidelines by Hong Kong Observatory (32). Two additional protective behaviours to “rest and avoid overexertion when working or conducting activities outdoors” and to “get to cooler places when feeling unwell” were bivariately associated with decreased outdoor physical activity but removed from further analysis due to the conceptual similarity with the outcome of interest.

**Table S2. Bivariate analysis for change in PA during extreme temperatures (Chi-squared tests and T-tests)**

| Chi-squared tests | Decreased PA in 2016 extreme cold | | | Decreased PA in 2017 extreme heat | | | Increased PA in either extreme temperature event | | |
| --- | --- | --- | --- | --- | --- | --- | --- | --- | --- |
| **Variables** | X^2^ (df) | p-value | p < 0.25 | X^2^ (df) | p-value | p < 0.25 | X^2^ (df) | p-value | p < 0.25 |
| Gender | 5.687 (1) | 0.017 | # | 12.212 (1) | <0.001 | # | 0.004 (1) | 0.95 |  |
| Age | 1.465 (3) | 0.69 |  | 6.72 (3) | 0.081 | # | 19.654 (3) | <0.001 | # |
| Education | 2.817 (2) | 0.245 | # | 6.73 (2) | 0.035 | # | 6.5 (2) | 0.039 | # |
| District | 6.092 (2) | 0.048 | # | 2.093 (2) | 0.351 |  | 1.569 (2) | 0.456 |  |
| Income | 0.151 (2) | 0.927 |  | 2.427 (2) | 0.297 |  | 7.722 (2) | 0.021 | # |
| Marital status | 1.507 (2) | 0.471 |  | 7.232 (2) | 0.027 | # | 7.552 (2) | 0.023 | # |
| Occupation | 4.642 (5) | 0.461 |  | 17.029 (5) | 0.004 | # | 24.255 (5) | <0.001 | # |
| Live alone | 0.045 (1) | 0.832 |  | 3.177 (1) | 0.075 | # | 0.43 (1) | 0.512 |  |
| Housing | 2.407 (2) | 0.3 |  | 0.494 (2) | 0.781 |  | 4.355 (2) | 0.113 | # |
| Home ownership | 0.173 (1) | 0.677 |  | 2.808 (1) | 0.094 | # | 2.054 (1) | 0.152 | # |
| General Health | 4.2 (3) | 0.241 | # | 5.167 (3) | 0.16 | #^ | 4.045 (3) | 0.257 |  |
| Long-term medication | 0.007 (1) | 0.932 |  | 1.647 (1) | 0.199 | # | 1.522 (1) | 0.217 | # |
| Multimorbidity | 0.003 (2) | 0.998 |  | 3.862 (2) | 0.145 | # | 5.241 (2) | 0.073 | # |
| Cardiovascular | 9.235 (1) | 0.002 | # | 0.334 (1) | 0.563 |  | 1.902 (1) | 0.168 | # |
| Diabetes | 0.066 (1) | 0.797 |  | 5.426 (1) | 0.02 | # | 1.214 (1) | 0.271 |  |
| Hypertension | 3.24 (1) | 0.072 | # | 10.35 (1) | 0.001 | # | 2.392 (1) | 0.122 | # |
| Respiratory disease | 1.572 (1) | 0.21 | # | 0.184 (1) | 0.668 |  | 0.584 (1) | 0.445 |  |
| Chronic pain (e.g. arthritis) | 0.297 (1) | 0.586 |  | 1.858 (1) | 0.173 | # | 0.926 (1) | 0.336 |  |
| Cancer | 0.707 (1) | 0.401 |  | 0.347 (1) | 0.556 |  | 0.163 (1) | 0.687 |  |
| Hypercholesteremia | 0.009 (1) | 0.923 |  | 1.381 (1) | 0.24 | # | 0.11 (1) | 0.74 |  |
| Seasonal Health (winter) | 22.18 (2) | <0.001 | # | \ | \ | \ | 3.046 (2) | 0.218 | # |
| Seek medical treatment (winter) | 3.57 (1) | 0.059 | # | \ | \ | \ | 0.203 (1) | 0.653 |  |
| Awareness of Cold Weather Warning | 0.159 (1) | 0.69 |  | \ | \ | \ | 0.922 (1) | 0.337 |  |
| Awareness of today's min temp | 2.957 (1) | 0.086 | # | \ | \ | \ | 0.203 (1) | 0.653 |  |
| Protective behaviour: avoid exposure to cold wind | 7.912 (1) | 0.005 | # | \ | \ | \ | 5.76 (1) | 0.016 | # |
| Protective behaviour: use heating device | 3.073 (1) | 0.08 | # | \ | \ | \ | 0.147 (1) | 0.701 |  |
| Seasonal Health (summer) | \ | \ | \ | 6.314 (2) | 0.043 | # | 1.263 (2) | 0.532 |  |
| Seek medical treatment (summer) | \ | \ | \ | 4.212 (1) | 0.04 | # | 0.912 (1) | 0.34 |  |
| Awareness of Very Hot Weather Warning | \ | \ | \ | 5.256 (1) | 0.022 | # | 0.27 (1) | 0.603 |  |
| Awareness of today's max temp | \ | \ | \ | 0.124 (1) | 0.724 |  | 0.036 (1) | 0.849 |  |
| Protective behaviour: avoid staying under sun | \ | \ | \ | 5.17 (1) | 0.023 | # | 0.005 (1) | 0.944 |  |
| Protective behaviour: use AC | \ | \ | \ | 4.56 (1) | 0.033 | # | 1.063 (1) | 0.302 |  |
|  |  |  |  |  |  |  |  |  |  |
| **T-tests** | **Decreased PA in 2016 extreme cold** | | | **Decreased PA in 2017 extreme heat** | | | **Increased PA in either extreme temperature event** | | |
| **Variables** | **t-value (df)** | **p-value** | **p < 0.25** | **t-value (df)** | **p-value** | **p < 0.25** | **t-value (df)** | **p-value** | **p < 0.25** |
| Agree cold impacts health | -2.948 (429) | 0.003 | # | \ | \ | \ | 1.395 (429) | 0.164 | # |
| Agree cold impacts can be avoided | -0.627 (432) | 0.531 |  | \ | \ | \ | 0.158 (432) | 0.875 |  |
| Agree have adequate knowledge to cope with cold weather | 0.703 (432) | 0.482 |  | \ | \ | \ | 0.557 (432) | 0.578 |  |
| Sum of protective behaviours (winter) | -1.438 (433) | 0.151 | # | \ | \ | \ | -0.535 (433) | 0.593 |  |
| Agree heat impacts health | \ | \ | \ | -2.394 (432) | 0.017 | # | -0.087 (432) | 0.931 |  |
| Agree heat impacts can be avoided | \ | \ | \ | 0.361 (432) | 0.718 |  | -0.651 (432) | 0.515 |  |
| Agree have adequate knowledge to cope with hot weather | \ | \ | \ | -1.433 (431) | 0.152 | # | -0.34 (431) | 0.734 |  |
| Sum of protective behaviours (summer) | \ | \ | \ | -1.434 (433) | 0.152 | # | -1.642 (433) | 0.101 | # |

^Both General Health and Seasonal Health (winter/summer) were bivariately associated with decreased PA in 2016 extreme cold and 2017 extreme heat. Due to their similarity, Seasonal Health was chosen for the subsequent multivariable analysis.

**Table S3. Multivariable logistic regression for Decreased outdoor PA in 2016 extreme cold, full model results**

| Predictors |  | n (%) | Unadjusted OR (95% CI) | Adjusted OR (95%CI) | p-value | |
| --- | --- | --- | --- | --- | --- | --- |
| Gender | Male | 198 (46) | 1 | 1 |  |  |
|  | Female | 232 (54) | 1.6 (1.09, 2.35) | 1.77 (1.16, 2.70) | 0.008 | * |
| Age | <25 | 65 (15.1) | 1 | 1 |  |  |
|  | 25-44 | 101 (23.5) | 1.04 (0.56, 1.94) | 1.12 (0.57, 2.20) | 0.74 |  |
|  | 45-64 | 168 (39.1) | 0.82 (0.46, 1.47) | 0.88 (0.46, 1.66) | 0.68 |  |
|  | 65+ | 96 (22.3) | 0.78 (0.42, 1.47) | 0.63 (0.29, 1.36) | 0.24 |  |
| Education | Post-secondary | 154 (35.8) | 1 | 1 |  |  |
|  | Secondary | 221 (51.4) | 0.73 (0.48, 1.11) | 0.73 (0.46, 1.16) | 0.18 |  |
|  | Primary or below | 55 (12.8) | 1.05 (0.57, 1.92) | 0.96 (0.45, 2.03) | 0.91 |  |
| District | Kowloon | 139 (32.3) | 1 | 1 |  |  |
|  | HK Island | 68 (15.8) | 1.4 (0.78, 2.53) | 1.57 (0.82, 3.00) | 0.17 |  |
|  | New Territories | 223 (51.9) | 1.73 (1.12, 2.68) | 1.98 (1.23, 3.17) | 0.005 | * |
| Seasonal Health | Same | 301 (70) | 1 | 1 |  |  |
|  | Worse | 97 (22.6) | 2.99 (1.87, 4.79) | 3.03 (1.85, 4.98) | <0.001 | * |
|  | Better | 32 (7.4) | 1.04 (0.49, 2.19) | 1.06 (0.47, 2.36) | 0.90 |  |
| Cardiovascular disease | | 22 (5.1) | 4.01 (1.54, 10.45) | 6.55 (2.26, 18.94) | 0.001 | * |

Final model: N= 430, 178 reported decreased outdoor PA. Predicted 66.3%, Nagelkerke R^2^ 0.155; Variables excluded during the stepwise analysis: Hypertension, Respiratory disease, Seek medical treatment, Know today's minimum temperature, Agree cold impacts health, Avoid exposure to cold winds, Use heating devices, and Sum of protective behaviours

**Table S4. Multivariable logistic regression for Decreased outdoor PA in 2017 extreme heat, full model results**

| Predictors |  | n (%) | Unadjusted OR (95% CI) | Adjusted OR (95%CI) | p-value | |
| --- | --- | --- | --- | --- | --- | --- |
| Gender | Male | 187 (45.3) | 1 | 1 |  |  |
|  | Female | 226 (54.7) | 2.06 (1.37, 3.09) | 2.20 (1.41, 3.44) | 0.001 | * |
| Age | <25 | 55 (13.3) | 1 | 1 |  |  |
|  | 25-44 | 94 (22.8) | 0.87 (0.45, 1.67) | 0.84 (0.40, 1.75) | 0.64 |  |
|  | 45-64 | 160 (38.7) | 0.79 (0.43, 1.45) | 0.87 (0.43, 1.75) | 0.70 |  |
|  | 65+ | 104 (25.2) | 0.46 (0.24, 0.91) | 0.76 (0.33, 1.75) | 0.52 |  |
| Education | Post-secondary | 148 (35.8) | 1 | 1 |  |  |
|  | Secondary | 210 (50.8) | 0.86 (0.56, 1.31) | 0.90 (0.55, 1.47) | 0.68 |  |
|  | Primary or below | 55 (13.3) | 0.4 (0.2, 0.81) | 0.44 (0.19, 1.04) | 0.06 |  |
| Seasonal Health | Same | 320 (77.5) | 1 | 1 |  |  |
|  | Worse | 47 (11.4) | 2.02 (1.13, 3.63) | 2.41 (1.21, 4.77) | 0.012 | * |
|  | Better | 46 (11.1) | 1.43 (0.76, 2.67) | 1.43 (0.73, 2.81) | 0.303 |  |
| Hypertension | | 63 (15.3) | 0.35 (0.18, 0.68) | 0.38 (0.18, 0.82) | 0.013 | * |
| Awareness of VHWW | | 362 (87.7) | 2.22 (1.11, 4.45) | 2.47 (1.16, 5.26) | 0.019 | * |
| Use AC | | 382 (92.5) | 2.6 (1.05, 6.46) | 2.74 (0.98, 7.69) | 0.055 |  |
| Agree heat impacts health | | Mean 3.80 (SD = 1.44) | 1.18 (1.03, 1.36) | 1.19 (1.02, 1.40) | 0.028 | * |

Final model: N= 413, 147 reported decreased outdoor PA. Predicted 67.3%, Nagelkerke R^2^ 0.165; Variables excluded during the stepwise analysis: Marital status, Occupation, Live alone, Home ownership, Diabetes, Chronic pain, Hypercholesteremia, Multimorbidity, Long-term medication, Seek medical treatment, Agree have adequate knowledge to cope with hot weather, Avoid staying out in the sun, and Sum of protective behaviours

**Table S5. Multivariable logistic regression for Increased outdoor PA in either extreme temperature, full model results**

| Predictors |  | n (%) | Unadjusted OR (95% CI) | Adjusted OR (95%CI) | p-value | |
| --- | --- | --- | --- | --- | --- | --- |
| Gender | Male | 180 (45.3) | 1 | 1 |  |  |
|  | Female | 217 (54.7) | 1.02 (0.5, 2.09) | 1.08 (0.48, 2.44) | 0.86 |  |
| Age | <25 | 53 (13.4) | 1 | 1 |  |  |
|  | 25-44 | 94 (23.7) | 0.44 (0.18, 1.08) | 0.35 (0.13, 0.96) | 0.042 | * |
|  | 45-64 | 153 (38.5) | 0.17 (0.06, 0.46) | 0.12 (0.04, 0.36) | <0.001 | * |
|  | 65+ | 97 (24.4) | 0.15 (0.05, 0.48) | 0.08 (0.02, 0.34) | 0.001 | * |
| Education | Post-secondary | 143 (36) | 1 | Excluded^ |  |  |
|  | Secondary | 204 (51.4) | 1.43 (0.67, 3.04) |  |  |  |
|  | Primary or below | 50 (12.6) | 0 (0, .) |  |  |  |
| Housing | Private | 198 (49.9) | 1 | 1 |  |  |
|  | Public | 137 (34.5) | 0.37 (0.15, 0.92) | 0.29 (0.10, 0.81) | 0.019 | * |
|  | Subsidized | 62 (15.6) | 0.57 (0.19, 1.73) | 0.69 (0.22, 2.18) | 0.52 |  |
| Avoid exposure to cold winds | | 317 (79.8) | 0.41 (0.19, 0.87) | 0.35 (0.15, 0.83) | 0.017 | * |
| Sum of summer protective behaviours | | Mean 5.01 (SD= 1.51) | 1.24 (0.96, 1.6) | 1.39 (1.04, 1.87) | 0.028 | * |

Final model: N= 397, 31 reported increased outdoor PA. Predicted 92.7%, Nagelkerke R^2^ 0.194; Variables excluded during the stepwise analysis: Marital status, Income, Occupation, Home ownership, Seasonal winter health, Cardiovascular disease, Hypertension, Long-term medication, Multimorbidity, and Agree cold impacts health

^Education was excluded from the multivariable logistic regression since there were zero cases of increased physical activity among those with primary education or below.

**Table S6. Sensitivity analysis for Decreased outdoor PA in 2016 extreme cold**

| **Variables selected from bivariate analysis (p < 0.25)** | **Manuscript findings (Forward stepwise logistic regression)** | **Generalized Linear Model (full model)** | **Multi-model inference (best model by lowest AIC)** | **Multi-model inference (best model, adjusted for gender, age & education)** |
| --- | --- | --- | --- | --- |
| Gender | Required, ** | . (p < 0.1) | * | Required, * |
| Age | Required | x |  | Required |
| Education | Required | x |  | Required |
| District | ** | ** | ** | ** |
| Seasonal Health | *** | *** | *** | *** |
| Cardiovascular disease | *** | *** | *** | *** |
| Hypertension |  | . (p < 0.1) | * | . (p < 0.1) |
| Respiratory disease |  | x |  |  |
| Seek medical treatment |  | x |  |  |
| Know today's minimum temperature |  | x |  |  |
| Agree cold impacts health |  | x | x | . (p < 0.1) |
| Avoid exposure to cold winds |  | . (p < 0.1) | * | . (p < 0.1) |
| Use heating devices |  | x |  |  |
| Winter protective behaviours |  | x |  |  |

Significance codes: ‘***’ p < 0.001; ‘**’ p < 0.01; ‘*’ p < 0.05; ‘.’ p < 0.1; ‘x’ included in model but non-significant

**Table S7. Sensitivity analysis for Decreased outdoor PA in 2017 extreme heat**

| **Variables selected from bivariate analysis (p < 0.25)** | **Manuscript findings (Forward stepwise logistic regression)** | **Generalized Linear Model (full model)** | **Multi-model inference (best model by lowest AIC)** | **Multi-model inference (best model, adjusted for gender, age & education)** |
| --- | --- | --- | --- | --- |
| Gender | Required, ** | * | *** | Required, *** |
| Age | Required | x |  | Required |
| Education | Required | x |  | Required |
| Seasonal Health | * | * | * | * |
| Hypertension | * | * | *** | ** |
| Awareness of VHWW | * | * | ** | * |
| Use AC | . (p < 0.1) | x | * | . (p < 0.1) |
| Agree heat impacts health | * | . (p < 0.1) | . (p < 0.1) | * |
| Marital status |  | x |  |  |
| Occupation |  | . (p < 0.1) Students and Service workers |  |  |
| Live alone |  | x |  |  |
| Home ownership |  | x | x | x |
| Diabetes |  | x |  |  |
| Chronic pain |  | x | x | x |
| Hypercholesteremia |  | x |  |  |
| Multimorbidity |  | x |  |  |
| Long-term medication |  | x |  |  |
| Seek medical treatment |  | x |  |  |
| Agree have adequate knowledge to cope with hot weather |  | x | x | x |
| Avoid staying out in the sun |  | x |  |  |
| Summer protective behaviours |  | x |  |  |

Significance codes: ‘***’ p < 0.001; ‘**’ p < 0.01; ‘*’ p < 0.05; ‘.’ p < 0.1; ‘x’ included in model but non-significant

**Table S8. Sensitivity analysis for Increased outdoor PA in either extreme temperature event**

| **Variables selected from bivariate analysis (p < 0.25)** | **Manuscript findings (Forward stepwise logistic regression)** | **Generalized Linear Model (full model)** | **Multi-model inference (best model by lowest AIC)** | **Multi-model inference (best model, adjusted for gender, age & education)** |
| --- | --- | --- | --- | --- |
| Gender | Required | x |  | Required |
| Age | Required, ** | x | *** | Required, *** |
| Housing | * | x | * | * |
| Avoid exposure to cold winds | * | * | ** | ** |
| Summer protective behaviours | * | * | * | * |
| Marital status |  | x |  |  |
| Income |  | x |  |  |
| Occupation |  | * Student |  |  |
| Home ownership |  | x |  |  |
| Seasonal winter health |  | x |  |  |
| Cardiovascular disease |  | x |  |  |
| Hypertension |  | x |  |  |
| Long-term medication |  | x |  |  |
| Multimorbidity |  | x |  |  |
| Agree cold impacts health |  | x |  |  |

Significance codes: ‘***’ p < 0.001; ‘**’ p < 0.01; ‘*’ p < 0.05; ‘.’ p < 0.1; ‘x’ included in model but non-significant

^Education was excluded since there were zero cases of increased physical activity among those with primary education or below.
